# Improving Flame Retardant Properties of Aliphatic Polyketone (POK)-Based Composites

**DOI:** 10.1021/acsomega.2c08066

**Published:** 2023-03-01

**Authors:** Cansu Yıldız, Yoldas Seki, Merve Ekti, Sibel Aker, Berkay Metin Leskeri, Mehmet Sarikanat, Lutfiye Altay

**Affiliations:** † The Graduate School of Natural and Applied Sciences, Dokuz Eylul University, Buca, İzmir 35390, Turkey; ‡ Faculty of Science, Dokuz Eylul University, Buca, Izmir 35160, Turkey; § İzmir Eğitim Sağlık Sanayi Yatırım A.Ş., Turgutlu, Manisa 45400, Turkey; ∥ Mechanical Engineering Department, Ege University, Bornova, Izmir 35040, Turkey

## Abstract

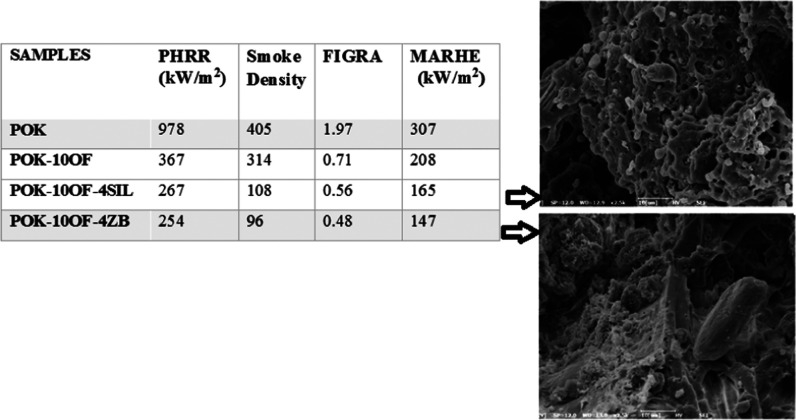

The effect of zinc borate (ZB) and high-molecular-weight siloxane (SIL) on flame retardancy, mechanical, and thermal properties of aliphatic polyketone (POK)-containing aluminum diethyl phosphinate (OF) was investigated in this study. Ten wt % OF is sufficient to obtain V0 rating according to the UL94 test. As the weight fraction of OF was increased, the flame retardancy properties and LOI values improved, while the tensile and impact properties decreased. To avoid the degradation in mechanical and impact properties as much as possible and obtain the same and better flame retardancy properties, synergists such as SIL and ZB were used. Flame retardancy of POK-based composites was determined by the limiting oxygen index (LOI) test, UL94 measurement, and cone calorimeter test. The additions of 1 wt % SIL and ZB have not led to a considerable decrease in the tensile strength and impact properties of POK-10OF. While ZB and SIL are very efficient in decreasing the smoke density, ZB is more efficient than SIL in increasing the LOI value of the composite. The addition of 1, 2, and 4 wt % ZB and SIL synergists did not lower their UL94 ratings. Moreover, it can be added that ZB is more efficient than SIL in decreasing the fire growth rate (FIGRA) and maximum average rate of heat emission (MARHE) values. Using OF (10 wt %) and ZB (4 wt %), LOI values higher than 32% and smoke density values lower than 150 were obtained.

## 1 Introduction

Thermoplastic materials are used in all areas of our lives, such as white goods, automobile, furniture, electrical devices, electronics, etc.^1^ Thermoplastic materials add different properties according to their usage areas. These features cover concepts such as safety, convenience, ergonomics, cost, and high mechanics.^2^ Thermoplastics are materials with a high tendency to burn due to their natural properties. Today, it has become possible to add flame retardant properties to thermoplastic materials with the technology that has developed over the years.^3^ Safety problems in the vehicle industry are tried to be solved by thermoplastic materials with flame retardant properties.

Safety in rail transportation systems is important for both passengers and valuable cargo. Fire safety is of great importance for rail systems. The safety risk can be reduced by choosing materials suitable for fire safety for trains. In addition, accidents can be prevented before they happen.^4^ According to EN 45545:2013, for the hazard level HL3, limiting oxygen index values must be min 32% for interiors (R22) and exteriors (R23) of railway vehicles. In addition, smoke density values must be a maximum of 150 and 300 for R22 and R23, respectively.

Polyamide is used in various applications in the automotive industry such as door handles, body/chassis/structure, seat components, safety restraints, engine esthetic under-the-hood covers and pulley covers, air/fuel/oil (air inlet manifolds), front-end modules, and cables and cable conduits in rail, and railway tracks.^5^ However, polyamide-based composite materials developed have certain disadvantages. High dehumidification capacity, deterioration in mechanical values over time, and changes in dimensional stability can be listed as the most important of these disadvantages.^6^ Studies on new materials that are more advantageous and alternative to the widely used thermoplastic materials are continuing.^7^

It is known that aliphatic polyketone (POK) is a polymer produced from olefin monomers and carbon monoxide (CO). Fundamental patents on catalysts and composition emerged in the early 1970s. However, these early resins could no longer be processed due to the catalyst. In 1996, Shell commercialized a terpolymer of carbon monoxide, ethylene, and small amounts of propylene under the trade name CARILON.^8^ POK exhibits very high impact strength, acceptable abrasion resistance, chemical resistance, and low gas permeability. In addition, the consumption of CO during POK production contributes to the world′s air pollution removal.^9^ Moreover, POK has the potential to be identified as a new environmentally friendly and high-performance engineering plastic. In addition, due to its chemical properties, POK is superior to polyamides in terms of flame retardant properties.^10^

To satisfy the European fire protection norm EN 45545:2013 for railway vehicles, flame retardant POK composites were prepared. In this study, the restrictions about LOI and smoke density values for the interiors (R22) and exteriors (R23) of railway vehicles have been tried to be met. Toward this aim, halogen-free, flame retardant, POK-based composite materials were prepared using aluminum diethyl phosphinate as a base FR additive. To improve the flame retardancy performance, synergists such as zinc borate and high-molecular-weight siloxane were used. The effect of synergists on UL94, the fire growth rate (FIGRA), the maximum average rate of heat emission (MARHE), and peak heat release rate (PHRR) were also investigated.

## 2 Materials and Methods

### 2.1 Materials

POK M330 was supplied from HYOSUNG Chemical Corporation. Aluminum diethyl phosphinate (Exolit OP 1230, OF), was purchased from Clariant. Synergistic agents, a masterbatch of super-high-molecular-weight siloxane Silmaprocess AP1142A (SIL) and zinc borate (ZB), ZB 467, were supplied from Silmaprocess and Ucgen Pigments & Polymer Additives, respectively.

### 2.2 Compounding Process

A compounding process was conducted using a twin-screw extruder (Leistritz Extruder Corporation Model ZSE 27 MAXX). After passing through the extruder die and water bath, polymer strands were cut into pellets using a pelletizer. Test specimens were obtained from composite granules by molding with an injection molding machine (Bole model BL90EK).

### 2.3 Mechanical Properties

Tensile properties of POK and its composites were determined by a tensile test conducted using a Hegewald & Peschke Inspect 20 universal testing machine equipped with a video extensometer system at room temperature according to the ISO 527 standard with a crosshead speed of 50 mm/min.

### 2.4 Impact Strength

Impact tests were conducted using a Instron tester according to the ISO 180 standard. The notched and un-notched impact strength values were obtained by receiving the average values of five samples.

### 2.5 SEM Analysis

SEM observations of the tensile fracture surface of the specimens of POK and its composites were conducted using SEM, COXEM, and EM-30 Plus operated at 10 kV. Before SEM analysis, gold was deposited on the surface of the specimens via a plasma sputtering apparatus.

### 2.6 Differential Scanning Calorimetry (DSC) Analysis

Crystallization and melting behaviors of POK and its composites were investigated with a differential scanning calorimeter (TA Instruments Q20 DSC). The samples were heated from 10 to 300 °C at a rate of 10 °C/min under a N_2_ gas atmosphere and cooled to −80 °C at the same rate after 3 min of isothermal holding at this temperature. In the last stage, it was heated from −80 to 300 °C with a heating rate of 10 °C/min.

### 2.7 Thermogravimetric Analysis (TGA)

The thermal stability of POK and its composites was determined by thermogravimetric analysis. TGA was carried out using a TG analyzer (TA Instruments Q50 TGA) by heating from room temperature to 800 °C with a heating rate of 10 °C/min under a N_2_ gas atmosphere to prevent oxidation effects.

### 2.8 Thermomechanical Analysis (TMA)

Thermomechanical analyses of POK and its composite materials were conducted using a thermomechanical analyzer (TA Instruments TMA Q400), and thermal expansion coefficients were determined. TMA was performed in the expansion mode. Samples with a size of 10 mm × 8 mm × 4 mm were heated from −30 to 120 °C with a heating rate of 5 °C/min under a load of 0.02 N.

### 2.9 Flammability Tests

The vertical burning tests for the specimen dimensions of 125 × 13 × 3 mm^3^^3^ were conducted according to UL94 standard using an ATLAS horizontal and vertical burning tester. Limiting oxygen index (LOI) values of samples were carried out according to the ISO 4589 standard with an LOI instrument (Fire Testing Technology). The flammability of the samples was determined according to ISO 5660 using an external heat flux of 50 kWm^–2^ with a dimension of 100 × 100 × 3 mm^3^. The examined flammability parameters are FIGRA, effective heat combustion (EHC), MARHE, time of peak heat release rate (tPHRR), PHRR, smoke density, and total heat release (THR) values. Smoke density measurements were carried out according to the ISO 5659 standard using a smoke chamber.

## 3 Results and Discussion

### 3.1 Tensile Properties

**Figure 1:**
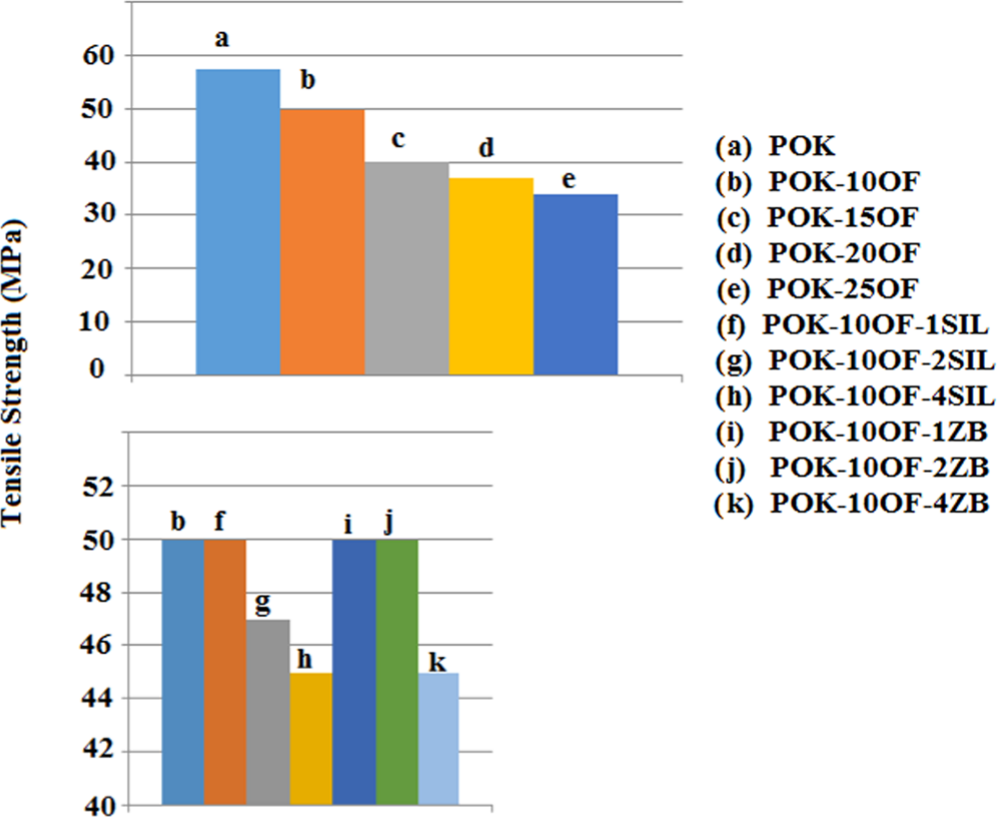
Tensile strength of POK and its composites.

The variations of the tensile strength of POK and its composites are presented in Figure 1. As can be seen from Figure 1a–e, OF addition into POK resulted in decreases in the tensile strength values of samples. Adding more OF into POK led to more decrease in tensile strength values. It is known that at a higher filler content, the interaction between the filler materials and polymer matrix was impeded, which leads to lower strength values.^11^ The effect of synergist addition together with OF on the tensile strength can be seen in Figure 1b–k. The addition of 1 wt % SIL and ZB has not led to a considerable decrease in the tensile strength of POK-10OF. However, 2 wt % of SIL caused a more significant decrease in the tensile strength than that of ZB. When the synergists were added by 4 wt %, SIL and ZB decreased the tensile strength by 10%. It is known that the tensile strength of a particle-filled polymer composite is strongly related to the interfacial adhesion developed between the filler and polymer matrix. If the filler–polymer adhesion is weak, the bond may be broken when the load is applied.^12^

**Figure 2:**
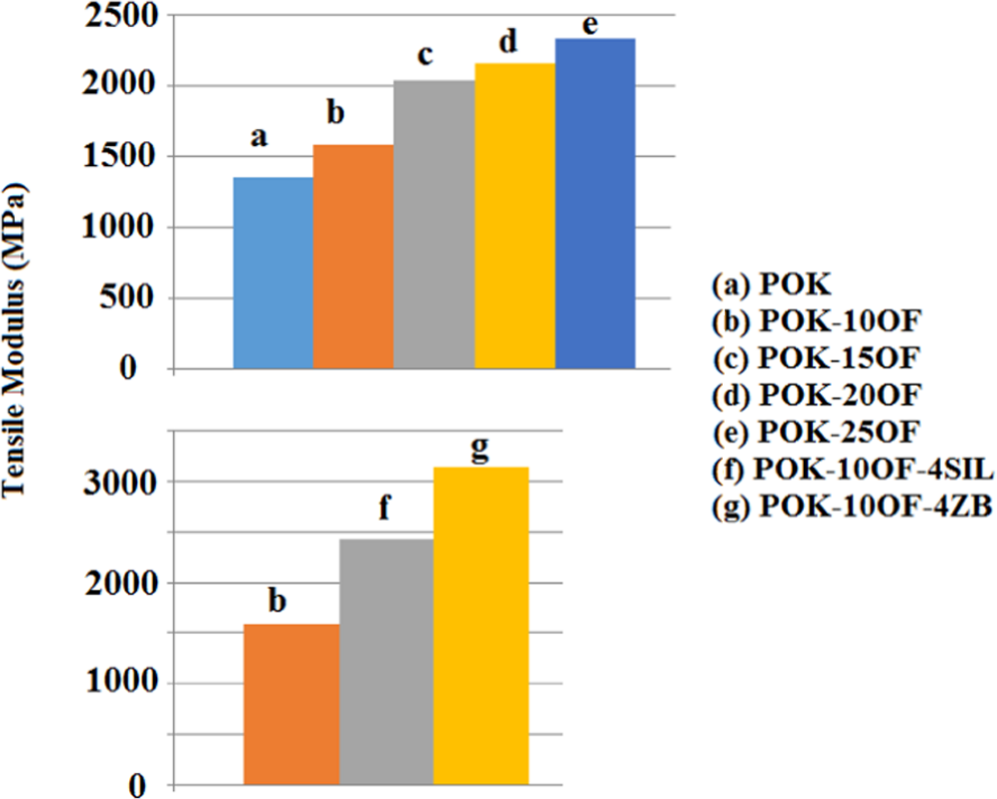
Tensile modulus values of POK and its composites.

The variations of the tensile modulus of POK and its composites are given in Figure 2. It is seen that 10, 15, 20, and 25 wt % OF additions into POK increased the tensile modulus of POK by 17, 50, 60, and 72%, respectively. The effect of synergists at a weight fraction of 4 wt % on the tensile modulus of POK-10OF can be seen in Figure 1b,f,g,h. The addition of ZB increased the tensile modulus values of POK-10OF. It can be noted that the greatest tensile modulus value was obtained by adding 4 wt % ZB. Incorporation of harder materials, compared to the polymer matrix, such as ZB into the polymer matrix led to higher tensile modulus values.^13^

### 3.2 Impact Strength

**Figure 3:**
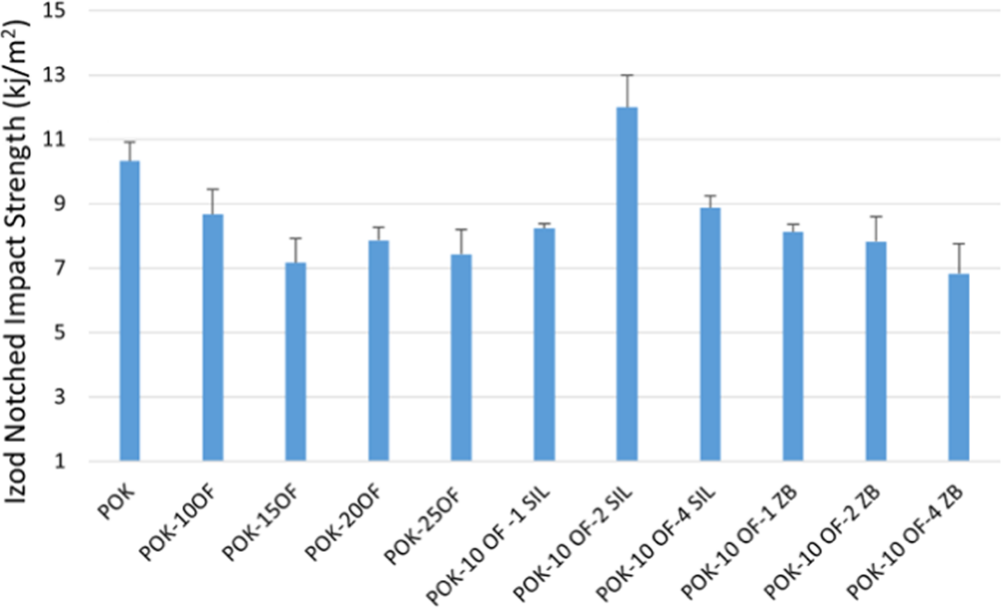
Izod notched impact strength of samples.

In general, when powder additives are added to polymer composites, it affects the mechanical values negatively. This is due to agglomeration of the flame retardant additive, poor interfacial interactions, imperfections, and voids.^14^ Izod notched impact strengths of POK and POK-based composites containing flame retardant additives are given in Figure 3. When 10%, 15%, 20%, and 25 wt % flame retardant additives were added to the POK composites, it did not exhibit a noticeable effect on the Izod notched impact strength. The impact strength of the POK composite containing 10 wt % OF was obtained relatively higher than the other ratios. For this reason, 1%, 2%, and 4 wt % SIL and zinc borate as synergist materials were added to the POK composite group containing 10 wt % OF, and their effect on mechanical values was investigated. Impact values were positively affected by adding 2 wt % SIL to POK composites containing flame retardant additives. The addition of zinc borate did not have a positive effect on the impact values of POK composites containing 10 wt % OF.

### 3.3 DSC Analysis

**Figure 4:**
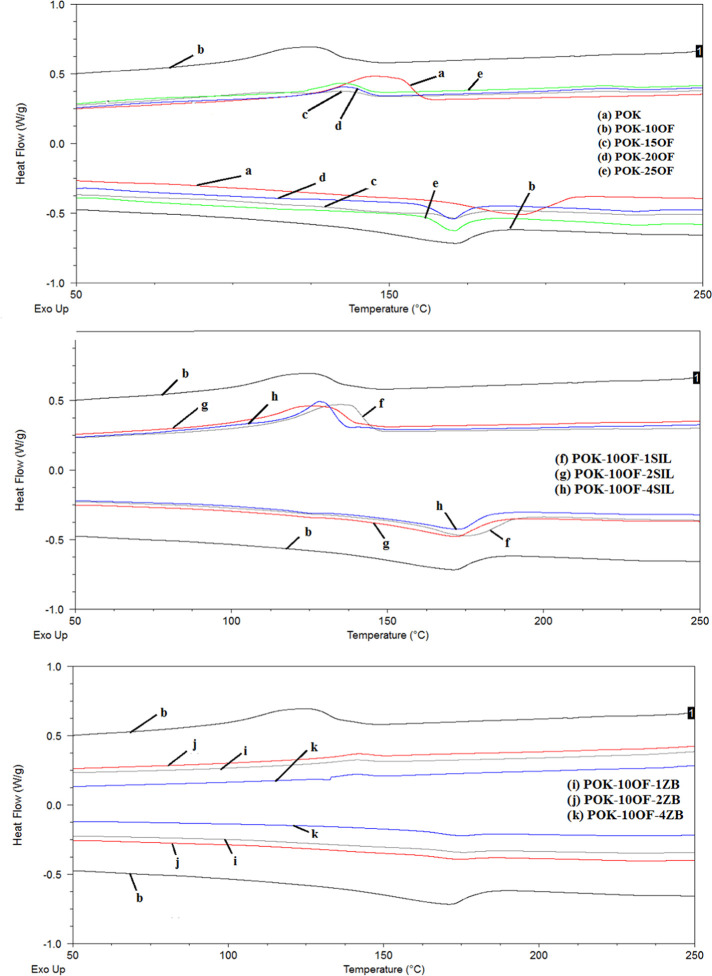
DSC curves of samples.

DSC curves of samples are shown in Figure 4, and the data obtained from Figure 4 are summarized in Table 1. The melting temperature (*T*_m_), crystallization temperature (*T*_c_), and melting enthalpy (Δ*H*_m_) of the samples were observed with DSC analysis, and the degree of crystallinity (*X*_c_) was calculated for each sample.^15^ The degree of crystallinity (*X*_c_) of the composites was calculated from the enthalpy of melting (Δ*H*) according to eq 1
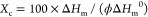
1where ϕ is the weight fraction of POK in the composite, and Δ*H*_m_^0^ is the melting enthalpy of the 100% crystalline POK polymer, reported as 227 J/g.^16^

**Table 1:** DSC Data of POK and Its Composites

Sample	*T*_m_ (°C)	*T*_c_ (°C)	Δ*H*_m_ (J/g)	*X*_c_ (%)
POK	193	145	26.2	11.5
POK-10OF	171	125	17.7	0.10
POK-15OF	171	134	7.3	0.04
POK-20OF	171	135	4.8	0.03
POK-25OF	171	134	5.8	0.03
POK-10OF-1SIL	175	135	28.8	0.14
POK-10OF-2SIL	172	125	27.3	0.14
POK-10OF-4SIL	173	128	17.4	0.09
POK-10OF-1ZB	173	140	1.7	0.01
POK-10OF-2ZB	172	149	1.9	0.01
POK-10OF-4ZB	172	140	1.5	0.01

According to the literature data, the melting temperature for aliphatic polyketone polymers was found to be around 200 °C.^17^ The melting point of the polyketone polymer (POK) used in the study was measured as 193 °C, the crystallization temperature was 145 °C, and the Δ*H*_m_ was measured as 26.2 J/g.

Melting temperatures of POK-10OF, POK-15OF, POK-20OF, and POK-25OF were measured as 171 °C. Polymer composites with POK-10OF and SILMA/ZB additives at different weight fractions were studied, and the effect of additives on the melting temperature was evaluated. SILMA and ZB additives at all added proportions tended to increase the melting temperature of POK-10OF.

### 3.4 Thermogravimetric Analysis (TGA)

**Figure 5:**
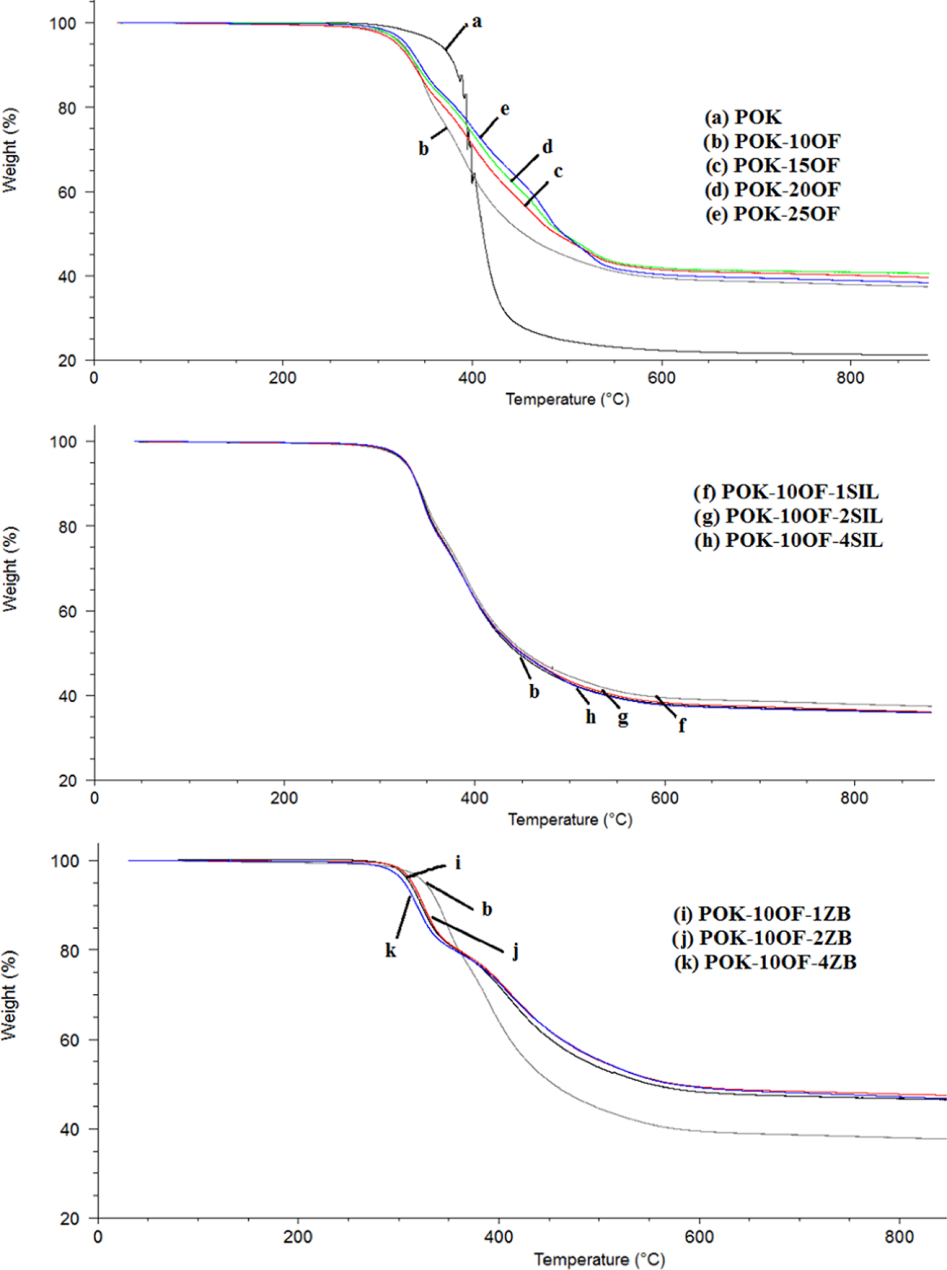
TGA curves of samples.

TGA curves of samples are presented in Figure 5, and the data obtained from Figure 5 are summarized in Table 2. Considering the degradation temperatures of POK-10OF, POK-15OF, POK-20OF, and POK-25OF, the highest degradation temperature was achieved with POK-10OF. Polymer composites with the POK-10OF base and SIL and ZB added at different weight fractions were studied and their decomposition temperatures were evaluated. SIL did not significantly change the decomposition temperature of POK-10OF; however, the ZB addition caused a decrease in the decomposition temperature of POK-10OF by about 20–30 °C. This is because the heat absorption capacity for ZB is lower than that for POK. When the weight ratio of ZB is increased, the threshold energy to initiate the degradation process is reached at lower temperatures, since ZB absorbs less heat in the composite.^18^

**Table 2:** TGA Data of Samples

Sample	*T* (°C) at %5 mass loss	*T*_max_ (°C)	Mass loss up to 800 °C (%)
POK	364	379	79
POK-10OF	326	348	62
POK-15OF	320	342	60
POK-20OF	321	342	59
POK-25OF	329	343	61
POK-10OF-1SIL	327	344	64
POK-10OF-2SIL	328	344	63
POK-10OF-4SIL	327	344	64
POK-10OF-1ZB	311	323	53
POK-10OF-2ZB	314	325	52
POK-10OF-4ZB	312	319	53

When the mass losses occurring in the period up to 800 °C are evaluated, the mass losses for POK-10OF, POK-15OF, POK-20OF, and POK-25OF are 62, 60, 59, and 61%, respectively. The mass loss was determined as 79% for POK. The addition of SIL and ZB additives significantly reduced the mass loss of the polymer. When the temperatures at the point of 5% mass loss were evaluated, ZB addition led to lower temperatures. However, SIL addition did not change considerably the temperature at 5% mass loss. It can be said that the thermal stability of the polymer decreased by the addition of FR materials according to the temperatures measured at mass losses and 5% mass loss.^19^

### 3.5 Thermomechanical Analysis (TMA)

The dimension change versus temperature was determined by TMA using the expansion mode. Thermal expansion coefficient (CTE) values of composites are given in Table 3. It was observed that the CTE value of POK decreased by adding FR materials. The decrease in the CTE value can be interpreted as an improvement in the dimensional stability of the polymer.^20^ Likewise, it has been observed that SIL and ZB additives in all ratios are used to improve the dimensional stability of the polymer.

**Table 3:** CTE Values of Samples

Sample	CTE (μm/m °C)
POK	99.5
POK-10OF	92.6
POK-15OF	96.2
POK-20OF	95.9
POK-25OF	96.2
POK-10OF-1SIL	85.8
POK-10OF-2SIL	72.4
POK-10OF-4SIL	92.1
POK-10OF-1ZB	92.3
POK-10OF-2ZB	84.8
POK-10OF-4ZB	86.6

### 3.6 Flame Retardancy Properties of POK-Based Composites

The parameters obtained from the cone calorimeter are collected in Table 4. It is known that PHRR is the most important and useful parameter with regard to assessing fire hazards.^21,22^ PHRR values of POK decreased by 62, 65, 70, and 73% with the addition of 10, 15, 20, and 25 wt % of OF, respectively. Considering PHRR values, the synergist ZB is more efficient than SIL to decrease the PHRR value. The reduced values of PHRR can be explained by the decomposition of flame retardant additives and the formation of a protective glassy char layer over the sample surface, during the combustion process.^23^

**Table 4:** Flame Retardant Properties of Composites

Sample	pHRR (kW/m^2^)	smoke density	THR (MJ/m^2^)	EHC (MJ/kg)	FIGRA^a^	MARHE (kW/m^2^)
POK	978	405	109	22.3	1.97	307
POK-10OF	367	314	98	19.7	0.71	208
POK-15OF	342	286	91	19.2	0.69	195
POK-20OF	298	274	88	19.1	0.65	181
POK-25OF	267	267	81	18.7	0.62	176
POK-10OF-1SIL	317	205	90	19.3	0.63	197
POK-10OF-2SIL	296	157	87	18.9	0.59	181
POK-10OF-4SIL	267	108	82	18.3	0.56	165
POK-10OF-1ZB	294	185	87	19.1	0.59	167
POK-10OF-2ZB	282	142	85	18.2	0.52	153
POK-10OF-4ZB	254	96	79	17.9	0.48	147

a FIGRA is calculated by dividing the peak HRR by the time taken to reach the peak HRR.

It is known that the smoke density (SD) is considered an important parameter to evaluate the performance of smoke suppression, and a low SD value indicates a high performance of smoke suppression.^24^ Smoke density values of POK, POK-10OF, POK-15OF, POK-20OF, POK-25OF, POK-10OF-1SIL, POK-10OF-2SIL, POK-10OF-4SIL, POK-10OF-1ZB, POK-10OF-2ZB, and POK-10OF-4ZB are 405, 314, 286, 274, 267, 205, 157, 108, 185, 142, and 96, respectively. OF, SIL, and ZB addition into POK decreased the total smoke value of POK. The lowest smoke density value was obtained using ZB as a synergist. ZB is decomposed into ZnO and B_2_O_3_, and ZnO may take part in the chemical reaction during combustion and can promote the formation of char and create a more cross-linking network in the char.^24^ In other words, ZB increases the cross-linking network in the char layer.^25^ It can be said that the effective char formation results in excellent smoke suppression^24^ and ZB addition led to lower smoke density values.

Other indices for fire hazards of the POK-based composites are FIGRA (equal to the value of PHRR/time to PHRR) and MARHE.^26^ From Table 4, 10 wt % OF addition into POK decreased MARHE and FIGRA values by 32 and 64%, respectively. When POK-10OF-4SIL and POK-10OF-4ZB are considered, it is seen that ZB leads to lower FIGRA and MARHE values. One can note that ZB is more efficient than SIL to decrease FIGRA and MARHE values. The THR is the integral of the HRR during the cone test and corresponds to the radiant flux levels.^27^ 10 wt % OF addition into POK decreased THR values by 10% and adding more OF led to lower THR values. With the addition of SIL and ZB, lower THR values were obtained. Moreover, ZB is more efficient than SIL in terms of the THR value. The EHC is the heat released from the combustion of the volatile portion of the material and is obtained by dividing the heat release rate by the mass loss rate. The higher smoke production and lower EHC value indicated that noncombustible gases exist in the gas phase;^22^ 10% OF addition into POK decreased the EHC value by about 12% and adding more OF led to lower EHC values.

Table 5 shows the LOI and UL94 flame ratings of samples for a thickness of 3 mm. The neat POK has a HB burning class according to the UL94 standard. When a 10 wt % OP-based flame retardant additive was added to the neat POK, the flame rating reached a V0 rating class. Formation of specific phosphorus species by means of the degradation reaction of phosphinates makes the char layer covered on the residual sample surface to inhibit further fusion and combustion.^14^ OF acted mainly through flame inhibition and phosphorus was released to the gas phase and induced the char formation in the residue.^28^ While the FR ratio was increased up to 25 wt %, the V0 flame rating of POK compounds did not change for the studied thickness. The LOI value of POK was obtained to be 21.0. When 10, 15, 20, and 25 wt % OF were added to POK, the LOI values increased to 29.8, 36.7, 40.0, and 39.6%, respectively. Considering LOI and UL94 ratings, the FR ratio was selected as 10 wt %. To see the synergistic effect of ZB and SIL, 1, 2, and 4 wt % synergists together with 10 wt % FR composites have been produced. ZB and SIL addition did not lead to variations in the UL94 ratings for the studied thickness. However, LOI values increased considerably with the addition of ZB and SIL. LOI values of POK-10OF increased from 29.8 to 31.8 and 31.1% with the addition of 1 wt % of SIL and ZB, respectively. As can be seen from Table 5, adding a larger amount of SIL did not lead to an increase in the LOI values. However, the addition of 2 and 4 wt % of ZB increased the LOI values to 32.0 and 34.8%, respectively. One can note that ZB is more efficient than SIL to improve the LOI values. This result is correlated with the fact that ZB is more efficient than SIL to decrease the smoke density values. Since ZB is used as a flame retardant and smoke suppressant synergist, the combination of ZB with some flame retardant systems could enhance the char formation, which results in the improvement of flame retardancy.^25^ Moreover, it can be added that ZB is used as a high-efficiency smoke inhibition agent and reduces the average CO production^29^

**Table 5:** UL94 and LOI Values of Samples

Sample	LOI (%)	UL94 rating
POK	21.0	HB
POK-10OF	29.8	V0
POK-15OF	36.7	V0
POK-20OF	40.0	V0
POK-25OF	39.6	V0
POK-10OF-1SIL	31.8	V0
POK-10OF-2SIL	30.4	V0
POK-10OF-4SIL	30.3	V0
POK-10OF-1ZB	31.1	V0
POK-10OF-2ZB	32.0	V0
POK-10OF-4ZB	34.8	V0

### 3.7 SEM Analysis

**Figure 6:**
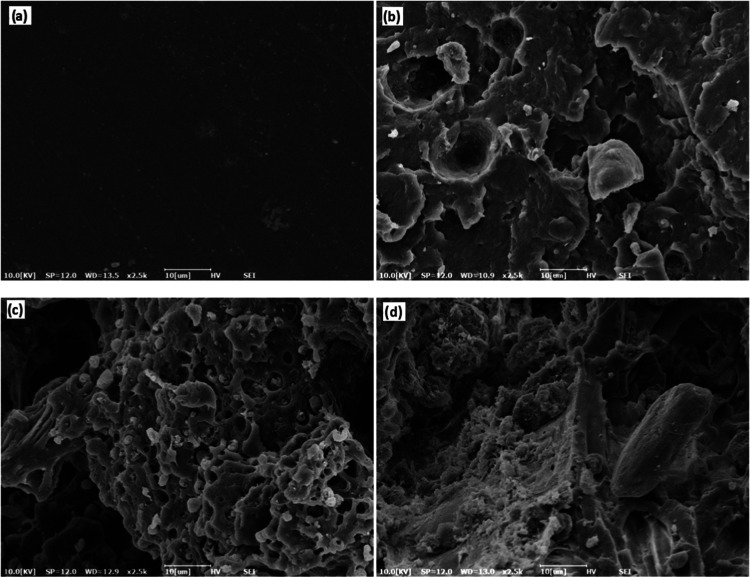
SEM images of samples: (a) POK, (b) POK-10OF, (c) POK-10OF-4SIL, and (d) POK-10OF-4ZB.

SEM images of POK and its composites are presented in Figure 6. As can be seen from Figure 6a, a continuous matrix structure can be seen. It could be clearly seen from Figure 6b that the particle size of OF was from about 1 to 10 μm. Pull-out particles that may indicate poor adhesion^30^ were observed. Since adhesion is weak, the particles pull away as the sample breaks. In Figure 6c, particles, which are in the range of about 800 nm to 3 μm, and holes from 1 to 3 μm, can be seen. Figure 6d shows that large particles are available within the structure. This may be attributed to the agglomeration of fine ZB particles.

## 4 Conclusions

In this study, the restrictions about LOI and smoke density values for the interiors (R22) and exteriors (R23) of railway vehicles according to the European fire protection norm EN 45545:2013 were met. Using OF (10 wt %) and synergists ZB (4 wt %), an LOI value higher than 32% and a smoke density value lower than 150 for the hazard level HL3 were obtained. The addition of 10 wt % OF into POK is sufficient to obtain a V0 rating according to the UL94 standard. However, to obtain higher LOI values, more OF or synergists such as ZB and SIL must be added to POK. Additionally, for lower smoke density, FIGRA, MARHE, and EHC values, instead of adding more OF, synergists such as ZB and SIL should be used. It can also be reported that ZB is more efficient than SIL to improve the LOI values and to decrease the smoke density, FIGRA, MARHE, PHRR, and THR values. On the basis of POK–OF composites, it can also be added that ZB is more efficient in smoke suppression than SIL. However, ZB led to lower Izod notched impact strength values than SIL. The addition of flame retardant materials (OF, ZB and SIL) decreased the tensile strength and thermal stability of POK. In addition, crystallinity of POK disappeared with the addition of OF (10, 15, 20, and 25 wt %) and ZB (1,2, and 4 wt %)/SIL (1, 2, and 4 wt %). In summary, lower amounts of synergists such as ZB and SIL are very efficient to improve flame retardancy properties of POK–OF composites without degrading the mechanical and impact properties as much as possible.

## References

[ref1] Reis J. P.; de Moura M.; Samborski S. Thermoplastic Composites and Their Promising Applications in Joining and Repair Composites Structures: A Review. Materials 2020, 13, 5832 10.3390/ma13245832. 33371418 PMC7767475

[ref2] Svensson; Shishoo N.; Gilchrist M. Manufacturing of Thermoplastic Composites from Commingled Yarns-A Review. J. Thermoplast. Compos. Mater. 1998, 11, 22–56. 10.1177/089270579801100102.

[ref3] Bourbigot S.; Duquesne S. Fire retardant polymers: recent developments and opportunities. J. Mater. Chem. 2007, 17, 2283–2300. 10.1039/B702511D.

[ref4] Jagadeesh P.; Puttegowda M.; Oladijo O. P.; Lai C. W.; Gorbatyuk S.; Matykiewicz D.; Rangappa S. M.; Siengchin S. A comprehensive review on polymer composites in railway applications. Polym. Compos. 2022, 43, 1238–1251. 10.1002/pc.26478.

[ref5] aRulkens R.; Koning C. 5.18 - Chemistry and Technology of Polyamides. In Polymer Science: A Comprehensive Reference, Matyjaszewski K.; Möller M., Eds.; Elsevier: 2012; 431–467.

[ref6] McKeen L. W. 1 - Introduction to Plastics and Polymers. In Film Properties of Plastics and Elastomers, 4th ed.; McKeen L. W., Ed.; William Andrew Publishing, 2017; pp 1–24.

[ref7] Levchik S. V.; Weil E. D. Flame retardancy of thermoplastic polyesters-a review of the recent literature. Polym. Int. 2005, 54, 11–35. 10.1002/pi.1663.

[ref8] Belov G.; Novikova E. Polyketones as Alternating Copolymers of Carbon Monoxide. Russ. Chem. Rev. 2004, 73, 267–291. 10.1070/RC2004v073n03ABEH000840.

[ref9] Ohsawa O.; Lee K.-H.; Kim B.-S.; Lee S.; Kim I.-S. Preparation and characterization of polyketone (PK) fibrous membrane via electrospinning. Polymer 2010, 51, 2007–2012. 10.1016/j.polymer.2010.02.045.

[ref10] Pilz G.; Guttmann P. Dynamic mechanical profile of polyketone compared to conventional technical plastics. AIP Conf. Proc. 2016, 1779, 070008 10.1063/1.4965540.

[ref11] Bigg D. M. Mechanical properties of particulate filled polymers. Polym. Compos. 1987, 8, 115–122. 10.1002/pc.750080208.

[ref12] Bishay I. K.; Abd-El-Messieh S. L.; Mansour S. H. Electrical, mechanical and thermal properties of polyvinyl chloride composites filled with aluminum powder. Mater. Des. 2011, 32, 62–68. 10.1016/j.matdes.2010.06.035.

[ref13] Fu S.-Y.; Feng X.-Q.; Lauke B.; Mai Y.-W. Effects of particle size, particle/matrix interface adhesion and particle loading on mechanical properties of particulate–polymer composites. Composites, Part B 2008, 39, 933–961. 10.1016/j.compositesb.2008.01.002.

[ref14] Guo S.; Pu S.; Zhao J.; Wang K.; Fu Q. Exploitation of a promising flame-retardant engineering plastics by molten composited polyketone and diethyl zinc phosphinate. Polym. Adv. Technol. 2019, 30, 1978–1988. 10.1002/pat.4630.

[ref15] Udayakumar M.; Kollár M.; Kristály F.; Leskó M.; Szabó T.; Marossy K.; Tasnádi I.; Németh Z. Temperature and Time Dependence of the Solvent-Induced Crystallization of Poly(l-lactide). Polymers 2020, 12, 1065 10.3390/polym12051065. 32384750 PMC7284506

[ref16] Cho S.; Lee J. S.; Jang H.; Park S.; An J. H.; Jang J. Comparative Studies on Crystallinity, Thermal and Mechanical Properties of Polyketone Grown on Plasma Treated CVD Graphene. Polymers 2021, 13, 919 10.3390/polym13060919. 33802662 PMC8002582

[ref17] Peters E. N. Engineering Thermoplastics-Materials, Properties, Trends. In Applied Plastics Engineering Handbook, 2nd ed.; Kutz M., Ed.; William Andrew Publishing, 2017; pp 3–26.

[ref18] Altay L.; Atagur M.; Akyuz O.; Seki Y.; Sen I.; Sarikanat M.; Sever K. Manufacturing of recycled carbon fiber reinforced polypropylene composites by high speed thermo-kinetic mixing for lightweight applications. Polym. Compos. 2018, 39, 3656–3665. 10.1002/pc.24394.

[ref19] Mohanty S.; Verma S. K.; Nayak S. K. Dynamic mechanical and thermal properties of MAPE treated jute/HDPE composites. Compos. Sci. Technol. 2006, 66, 538–547. 10.1016/j.compscitech.2005.06.014.

[ref20] Rukmini K.; Bommulu R.; Shetty S.; Taraiya A.; Bandyopadhyay S.; Hatna S. Development of Eco-Friendly Cotton Fabric Reinforced Polypropylene Composites: Mechanical, Thermal, and Morphological Properties. Advances in Polymer Technology 2013, 32 10.1002/adv.21327.

[ref21] Schartel B.; Hull T. R. Development of fire-retarded materials-Interpretation of cone calorimeter data. Fire Mater. 2007, 31, 327–354. 10.1002/fam.949.

[ref22] Hu Z.; Chen L.; Lin G.-P.; Luo Y.; Wang Y.-Z. Flame retardation of glass-fibre-reinforced polyamide 6 by a novel metal salt of alkylphosphinic acid. Polym. Degrad. Stab. 2011, 96, 1538–1545. 10.1016/j.polymdegradstab.2011.03.010.

[ref23] Formicola C.; De Fenzo A.; Zarrelli M.; Frache A.; Giordano M.; Camino G. Synergistic effects of zinc borate and aluminium trihydroxide on flammability behaviour of aerospace epoxy system. Express Polym. Lett. 2009, 3, 376–384. 10.3144/expresspolymlett.2009.47.

[ref24] Lu N.; Zhang P.; Wu Yn.; Zhu D.; Pan Z. Effects of Size of Zinc Borate on the Flame Retardant Properties of Intumescent Coatings. Int. J. Polym. Sci. 2019, 2019, 2424531 10.1155/2019/2424531.

[ref25] Feng C.; Zhang Y.; Liang D.; Liu S.; Chi Z.; Xu J. Influence of zinc borate on the flame retardancy and thermal stability of intumescent flame retardant polypropylene composites. J. Anal. Appl. Pyrolysis 2015, 115, 224–232. 10.1016/j.jaap.2015.07.019.

[ref26] Sacristán M.; Hull T. R.; Stec A. A.; Ronda J. C.; Galià M.; Cádiz V. Cone calorimetry studies of fire retardant soybean-oil-based copolymers containing silicon or boron: Comparison of additive and reactive approaches. Polym. Degrad. Stab. 2010, 95, 1269–1274. 10.1016/j.polymdegradstab.2010.03.015.

[ref27] Schartel B.; Kunze R.; Neubert D. Red phosphorus–controlled decomposition for fire retardant PA 66. J. Appl. Polym. Sci. 2002, 83, 2060–2071. 10.1002/app.10144.

[ref28] Braun U.; Schartel B. Flame Retardancy Mechanisms of Aluminium Phosphinate in Combination with Melamine Cyanurate in Glass-Fibre-Reinforced Poly(1,4-butylene terephthalate). Macromol. Mater. Eng. 2008, 293, 206–217. 10.1002/mame.200700330.

[ref29] Xu B.; Zhao S.; Shan H.; Qian L.; Wang J.; Xin F. Effect of two boron compounds on smoke-suppression and flame-retardant properties for rigid polyurethane foams. Polym. Int. 2022, 71, 1210–1219. 10.1002/pi.6403.

[ref30] Renner K.; Kenyó C.; Móczó J.; Pukánszky B. Micromechanical deformation processes in PP/wood composites: Particle characteristics, adhesion, mechanisms. Composites, Part A 2010, 41, 1653–1661. 10.1016/j.compositesa.2010.08.001.

